# Potential Use of Hyperspectral Reflectance as a High-Throughput Nondestructive Phenotyping Tool for Assessing Salt Tolerance in Advanced Spring Wheat Lines under Field Conditions

**DOI:** 10.3390/plants10112512

**Published:** 2021-11-19

**Authors:** Salah El-Hendawy, Nasser Al-Suhaibani, Muhammad Mubushar, Muhammad Usman Tahir, Yahya Refay, ElKamil Tola

**Affiliations:** 1 Department of Plant Production, College of Food and Agriculture Sciences, King Saud University, Riyadh 11451, Saudi Arabia; nsuhaib@ksu.edu.sa (N.A.-S.); mmubushar@ksu.edu.sa (M.M.); mtahir@ksu.edu.sa (M.U.T.); refay@ksu.edu.sa (Y.R.); 2 Department of Agronomy, Faculty of Agriculture, Suez Canal University, Ismailia 41522, Egypt; 3 Precision Agriculture Research Chair (PARC), College of Food and Agriculture Sciences, King Saud University, Riyadh 11451, Saudi Arabia; etola@ksu.edu.sa

**Keywords:** breeding, grain yield, multiple linear regression, spectral reflectance indices, stress tolerance indices, vegetation index, water index

## Abstract

The incorporation of stress tolerance indices (STIs) with the early estimation of grain yield (GY) in an expeditious and nondestructive manner can enable breeders for ensuring the success of genotype development for a wide range of environmental conditions. In this study, the relative performance of GY for sixty-four spring wheat germplasm under the control and 15.0 dS m^−1^ NaCl were compared through different STIs, and the ability of a hyperspectral reflectance tool for the early estimation of GY and STIs was assessed using twenty spectral reflectance indices (SRIs; 10 vegetation SRIs and 10 water SRIs). The results showed that salinity treatments, genotypes, and their interactions had significant effects on the GY and nearly all SRIs. Significant genotypic variations were also observed for all STIs. Based on the GY under the control (GYc) and salinity (GYs) conditions and all STIs, the tested genotypes were classified into three salinity tolerance groups (salt-tolerant, salt-sensitive, and moderately salt-tolerant groups). Most vegetation and water SRIs showed strong relationships with the GYc, stress tolerance index (STI), and geometric mean productivity (GMP); moderate relationships with GYs and sometimes with the tolerance index (TOL); and weak relationships with the yield stability index (YSI) and stress susceptibility index (SSI). Obvious differences in the spectral reflectance curves were found among the three salinity tolerance groups under the control and salinity conditions. Stepwise multiple linear regressions identified three SRIs from each vegetation and water SRI as the most influential indices that contributed the most variation in the GY. These SRIs were much more effective in estimating the GYc (R^2^ = 0.64 − 0.79) than GYs (R^2^ = 0.38 − 0.47). They also provided a much accurate estimation of the GYc and GYs for the moderately salt-tolerant genotype group; YSI, SSI, and TOL for the salt-sensitive genotypes group; and STI and GMP for all the three salinity tolerance groups. Overall, the results of this study highlight the potential of using a hyperspectral reflectance tool in breeding programs for phenotyping a sufficient number of genotypes under a wide range of environmental conditions in a cost-effective, noninvasive, and expeditious manner. This will aid in accelerating the development of genotypes for salinity conditions in breeding programs.

## 1. Introduction

Insufficient freshwater supplies for the agriculture sector require a parallel increase in the use of nonconventional water resources for the sustainable production of food crops. Since there are ample sources of saline water, several countries in arid and semiarid regions have embraced the use of saline water in the agricultural sector to support the shortage of freshwater resources and complement the irrigation water demand [1,2,3]. However, continuously irrigating crops with saline waters may lead to a significant reduction in their potential yield if the salinity levels exceed the plant tolerance limits. Salinity stress could reduce the potential yield of major agricultural crops by more than 50% [2,4,5]. Since bread wheat is moderately tolerant of salinity, this crop still loses more than 60% of its potential yield due to significant adverse impacts of salinity stress on their growth and development through ion toxicities, nutritional imbalance, and osmotic stress [6,7,8]. Therefore, when irrigating a wheat crop with saline water, it will be urgent to apply some feasible strategies in order to alleviate these adverse impacts of salinity stress.

Although there are numerous strategies are available for alleviating salinity stress, including a strategy of the exogenous application of nanoparticles, microelements, plant hormones, and growth regulators [9], the selection and development of new genotypes with high salt tolerance and maintaining an economic grain yield is still recognized as the most feasible and effective strategy for addressing this challenge [7,10]. The primary steps to enhance the salt tolerance of genotypes in breeding programs are to generate a large number of crossing lines and subsequently selecting within these lines during the evaluation process using several morphophysiological and biochemical plant traits as the screening criteria [11,12]. In the evaluation process, plant breeders often use the grain yield (GY) as the main screening criterion. Since GY is characterized by a low heritability and high environment by genotype interactions, the right decision for identifying superior lines with high GY too often needs to evaluate them in different environments and for several growing seasons. Small combine harvesters could be used to harvest experimental plots and measure the GY efficiently, but this method still remains laborious and expensive in terms of financial and time resources, particularly when it has to be done for a large number of genotypes that are usually evaluated by plant breeders. Additionally, it is difficult to use this method to measure the GY of crossing lines at the early generations of a breeding program, where the lines still have a small number of plants. Moreover, it is difficult to estimate the GY before the plants reach the physiological maturity stage. Thus, creating selection tools that are able to indirectly assess the GY for a sufficient number of lines at early growth stages in a rapid, routine, time- and cost-efficient, and nondestructive manner is urgently required in breeding programs.

Interestingly, during the different phenological growth stages, the potential GY of a crop under any growing conditions can be predicted through several integrative physiological traits, such as the photosynthetic area of the canopy, photosynthetic efficiency (PE), photosynthetically active radiation (PAR), leaf area index (LAI), vegetative vigor, crop dry matter (CDM), and the contents of the chlorophyll and water [13,14]. Therefore, several studies have reported that the characteristics of spectral reflectance by a crop canopy at specific regions of the electromagnetic spectrum are closely associated with the changes that take place in these physiological traits [15,16,17,18,19,20,21,22]. For example, a close relationship was found between the spectral reflectance at 760–1300 nm and the changes in the CDM and LAI in a winter wheat crop [23,24], between reflectance at 680 and 740 nm and the change in PE in a sunflower crop [25], and between spectral reflectance at 970, 1200, 1240, 1400, 1730, 1950, 2100, and 2250 nm and the changes in the leaf water status in different field crops [26,27,28]. Thus, more recent studies have suggested that GY can be estimated during different phenological growth stages using the spectral reflectance tool by simultaneously estimating the relevant crop traits that contribute to the GY. This tool can evaluate a large number of crossing lines or genotypes in a fast and nondestructive manner. Therefore, this tool can serve as a promising indirect selection criterion for breeding programs. Several spectral reflectance indices (SRIs), which are formulated using simple mathematical equations (e.g., differences and ratios) and spectral reflectance data at given wavelengths, have been developed for this purpose. In general, the different SRIs are usually developed based on their close relationships with different plant physiological traits, such as the photosynthetic capacity, CDM, pigment contents, chlorophyll fluorescence, LAI, transpiration rate, canopy water content, etc. [19,22,26,28,29,30,31,32]. For instance, the different normalized difference vegetation indices (NDVI, BNDVI, GNDVI, and RNDVI), which combine wavelengths from visible (VIS) and near-infrared (NIR) regions, were developed based on their relationship with the photosynthetic capacity, pigment content, and green biomass [22,33,34,35]. The different normalized water indices (NWI-1–4), which combine wavelengths from the NIR region [30] and water balance index (WABI), which combine wavelengths from VIS and shortwave-infrared (SWIR) regions [28,32], were developed based on their relationship with the water status of the canopy. The dry matter content index (DMCI) and normalized difference moisture index (NDMI) were also developed based on their strong relationship with the shoot dry weight, photosynthesis rate, and stomatal conductance of bread wheat under salinity stress conditions [19]. The different SRIs have been developed for several field crops under either normal or stress conditions. This reflects that there are several SRIs that could be used instead of the destructive selection criteria for breeding purposes as indirect selection criteria to differentiate genotypic differences in the GY at early phenological growth stages.

To date, several attempts have been made to evaluate the ability of using different vegetation SRIs and water SRIs as rapid and nondestructive screening criteria to differentiate genotypes for GYs under water-stressed and/or well-watered conditions in different field crops [15,16,36,37,38,39,40,41,42]. Some studies have reported that the SRIs, especially water SRIs, were effective at differentiating genotypes for the GY under water-stressed conditions, while they showed weak relationships with the GY under well-watered conditions; the opposite was true in other studies. Additionally, some studies have reported that the GY can be estimated at early phenological growth stages by SRIs, while other studies have mentioned that efficient SRIs for estimating the GY appeared at the late growth stages. Furthermore, several studies have evaluated the potential of SRIs as screening criteria using a small number of genotypes, which restricts the use of SRIs for breeding purposes [36]. This discrepancy among the results of studies requires further research. Besides, no results have been published until now to evaluate the potential of using different SRIs to indirectly estimate the GY for a large number of spring wheat lines under salinity stress conditions. Most of the studies regarding the use of SRIs as screening criteria for evaluating a large number of genotypes have been performed under normal condition or water and temperature stresses conditions. However, for salinity studies, the use of SRIs as screening criteria has been performed using a rather limited number of genotypes [19,22,26,35].

Generally, the performance of all genotypes for the grain yield is often not consistent across different levels of salinity stress [43]. Therefore, to select desirable genotypes that perform well under a wide range of stress levels, several stress tolerance indices (STIs) that are calculated in simple mathematical equations and reflect the performances of genotypes in both non-stress and stress conditions have been proposed. A common STI is the yield stability index (YSI), which assess the performances of genotypes for GYs under stress conditions relative to GYs under non-stress conditions. Therefore, this index indicates the amount of genetic resistance to stress where the genotypes with a high value of YSI perform well in both conditions [44]. Therefore, this index is widespread and has a broad relevance for evaluating the salt tolerance of genotypes [45,46]. The stress tolerance index (STI) is useful in identifying genotypes with high stress tolerance and high potential yields [47]. The absolute differences in the GY between stress and nonstress conditions is called the tolerance index (TOL). The higher values of this index indicate the susceptibility of a given genotype to stress [47]. The stress susceptibility index (SSI), proposed by Singh et al. [48], estimates the relative tolerance for GY reduction of a genotype relative to the average reduction of GY for all genotypes due to stress. Previous studies have reported that genotypes with a value of SSI greater than the unit are more tolerant to stress than those with SSI lower than the unit [49]. The geometric mean productivity (GMP estimates the performance of GYs of genotypes under stress and nonstress conditions, taking into consideration the variability in stress intensities over the years and in different environments [47]. Therefore, the different STIs can provide reliable indications to evaluate the performance of a genotype for a GY under either stress conditions, nonstress conditions, or both conditions. With the STIs, we can define the genotypes that exhibited good or weak performances in both conditions and good only in the stress condition or in nonstress condition [47].

As far as we are aware, this is the first report on spectrally evaluating the GYs and STIs of a large number of wheat genotypes under control and salinity conditions using SRIs. The main goal of this study was to evaluate the potential of using different SRIs as an indirect screening tool to rapidly and nondestructively assess the salt tolerance of a large number of wheat genotypes based on their relationships with the GY under control and salinity conditions and different STIs. The specific objectives were to (1) evaluate the impacts of two salinity levels on GY, STIs, and SRIs of advanced breeding wheat lines and commercial wheat cultivars; (2) classify the salt tolerance levels of the evaluated lines and cultivars based on GYs and STIs; (3) compare the spectral reflectance signatures of the canopy for different salinity tolerance groups; and (4) assess the potential of SRIs as rapid and nondestructive tools for detecting GYs and STIs of different salinity tolerance groups.

## 2. Results

### 2.1. Impact of Salinity Treatment, Genotype, Year, and Their Interactions on Grain Yield, Stress Tolerance Indices, and Spectral Reflectance Indices

Mean squares from the ANOVA analysis (Table 1) revealed that the salinity treatment (ST) main effect in each year and the combined analysis of two years was significant for the GY and all SRIs, except for one and five out of the 10 vegetation SRIs, as well as one and three out of the 10 water SRIs in the first year and second year, respectively. The genotype (G) main effect in each year and the combined analysis was significant for the GY, all SRIs, and all STIs. The ST by G interaction had a highly significant effect (*p* ≤ 0.001) on the GY and all SRIs, except for two out of 10 water SRIs in the second year (Table 1). The years’ (Y) main effect in the combined analysis was not significant for the GYs and all STIs, except for the SSI but was significant for almost all SRIs. The Y by ST interaction was not significant for the GY, two vegetation SRIs, and three water SRIs. The Y by G interaction was highly significant for GYs, all SRIs, and all STI, except for the STI and GMP (Table 1). The interaction effect between the ST, G, and Y was significant for all SRIs but not for the GY (Table 1).

### 2.2. Genotypic Performance in Grain Yield, Stress Tolerance Indices, and Spectral Reflectance Indices under Control and Salinity Conditions

**Figure 1:**
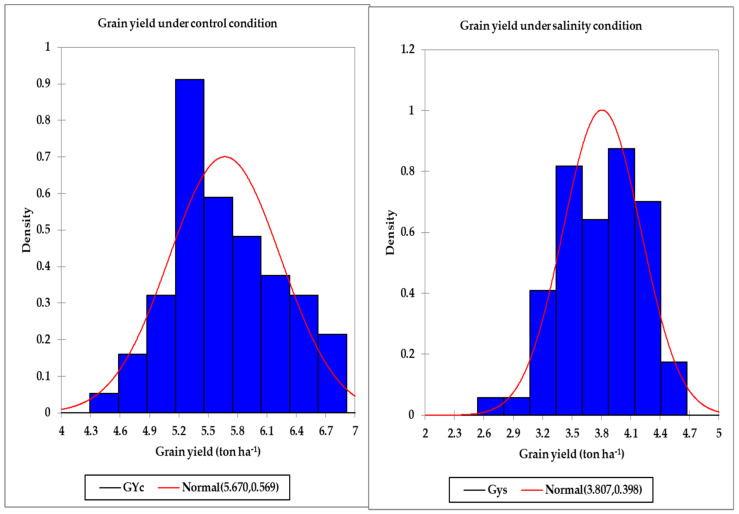
Distribution of the grain yield under the control and salinity conditions.

Table 2 displays the minimum, maximum, and mean values across all genotypes for the GY, SRIs, and STIs under the control and salinity conditions in both years. There was a wide range between the minimum and maximum values for the GY, all SRIs (except the BNDVI and WI), and all STIs under the control and salinity conditions in both years. In general, the maximum values were two–five times higher than the minimum values for the GY and all indices, which indicated broad genotypic differences for these traits under the control and salinity conditions (Table 2). For instance, the GYs across all genotypes ranged from 3.92 to 7.24 tons ha^−1^ and from 4.24 to 7.81 tons ha^−1^ under the control treatment and from 2.51 to 4.81 tons ha^−1^ and from 2.27 to 5.21 tons ha^−1^ under the salinity treatment in the first and second years, respectively (Table 2). Additionally, the histogram analysis for the GY as the average values of two years under the control and salinity conditions provided a very general view for the distribution of GY of the tested genotypes, which showed continuous variations for the GY under both conditions (Figure 1). Generally, the tested genotypes were normally distributed under both conditions. Regarding the SRIs, the mean values of the vegetation SRIs were, in general, higher for the control treatment than those for the salinity treatment, particularly in the first year. Similar trends were also observed for four out of the 10 water SRIs (WI, NDWI, NDMI, and SWSI-1) in both years; the opposite was true for the other water SRIs (Table 2).

### 2.3. Association of Grain Yield and Stress Tolerance Indices with Spectral Reflectance Indices across All Genotypes

**Figure 2:**
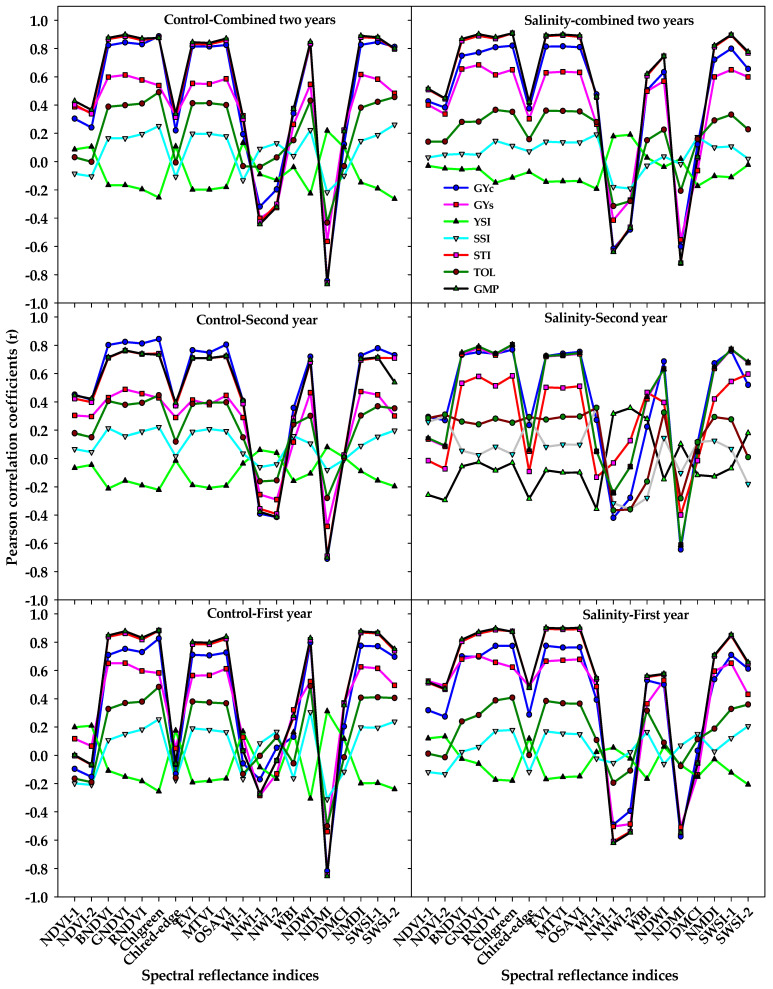
Correlation coefficients (r) of the relationships between different vegetation SRIs and water SRIs that were calculated from spectral measurements taken under both the control and salinity conditions and grain yield under the control (GYc), grain yield under salinity (GYs), and five stress tolerance indices (STIs) for each year and across two years. The r is significant at alpha = 0.05 when their values ≤−0.25 or ≥0.25. The full names of the abbreviations of five stress tolerance indices and different spectral reflectance indices are mentioned in Table 6.

The correlations of GYs and STIs with different SRIs that were calculated from spectral measurements taken under the control and salinity treatments for each year and combined two years are presented in Figure 2. In general, the different SRIs from both treatments did not show any significant correlations with the YSI and SSI, with only a few SRIs from the salinity treatment exhibiting a weak correlation (r = −0.36 − 0.36) with both indices at the second year (Figure 2). Seven out of the 10 vegetation SRIs from either the control or salinity treatments (BNDVI, GNDVI, RNDVI, Chlgreen, EVI, MTVI, and OSAVI) exhibited strong correlations with the GYc, STI, and GMP (r = 0.70 − 0.90); weak-to-moderate correlations with the GYs (r = 0.28 − 0.70); and weak correlations with the TOI (r = 0.25 − 0.49) in each year and combined two years. Five out of the 10 water SRIs (NDWI, NDMI, NMDI, SWSI-1, and SWSI-2) from the control treatment exhibited strong correlations with the GYc, STI, and GMP (r = 0.70 − 0.89), whereas they exhibited moderate-to-strong correlations with the same three traits (r = 0.50 − 0.90) when they calculated from the salinity treatment. At the first year and combined two years, the following water SRIs: WI, NWI-1, NWI-2, and WBI from the salinity treatment correlated better with the GYc, GYs, STI, and GMP than those calculated from the control treatment (Figure 2).

### 2.4. Grouping Genotypes Based on Their Salt Tolerance Level

**Figure 3:**
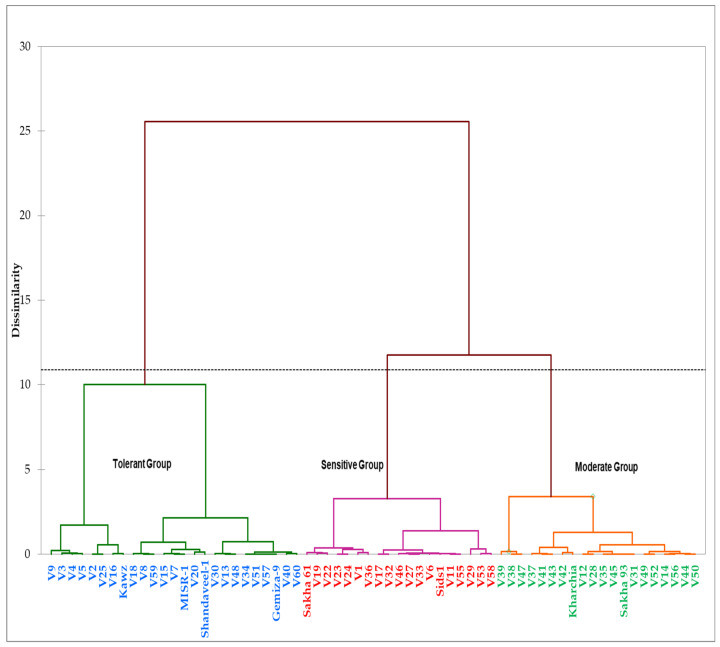
Hierarchical clusters analysis of the 64 genotypes derived by Ward’s methods and based on the grain yield under the control (GYc) and salinity (GYs) conditions and the five stress tolerance indices across two years.

**Figure 4:**
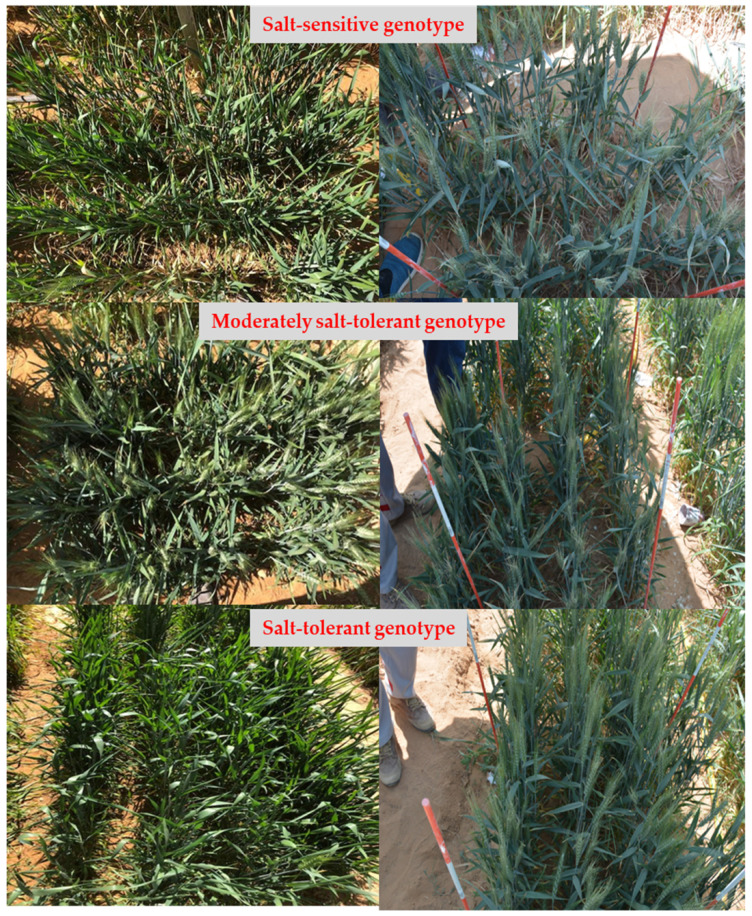
Photographs show examples of salt-tolerant, salt-sensitive, and moderately salt-tolerant genotypes at two different growth stages.

Based on grain yields of genotypes under the control (GYc) and salinity (GYs) conditions and four STIs (YSI, SSI, TOL, STI, and GMP) across two years, the genotypes were grouped into three distinct salinity tolerance groups (Figure 3). The first group contained four commercial cultivars (Kawz, Misr-1, Shandawel-1, and Gemiza-9) and 21 RILs. The genotypes of this group attained higher values for the GYc, GYs, STI, TOL, and GMP (Table 3). The second group included a salt-sensitive genotype (Sakha 61), moderately salt-tolerant genotype (Sids 1), and 17 RILs. These genotypes attained a lower value for the GYs, YSI, STI, and GMP and a higher value for the SSI. The genotypes in the third group, containing the two salt-tolerant genotypes (Kharchia 65 and Sakha 93) and 18 RILs, attained a higher value for the YSI; a lower value for the SSI and TOL; and a medium value for the GYs, STI, and GMP (Table 3). Based on these results, the genotypes in the first, second, and third groups could be classified as salt-tolerant, salt-sensitive, and moderately salt-tolerant genotypes, respectively (Figure 4).

### 2.5. Spectral Signatures of the Three Salinity Tolerance Groups under Control and Salinity Conditions

**Figure 5:**
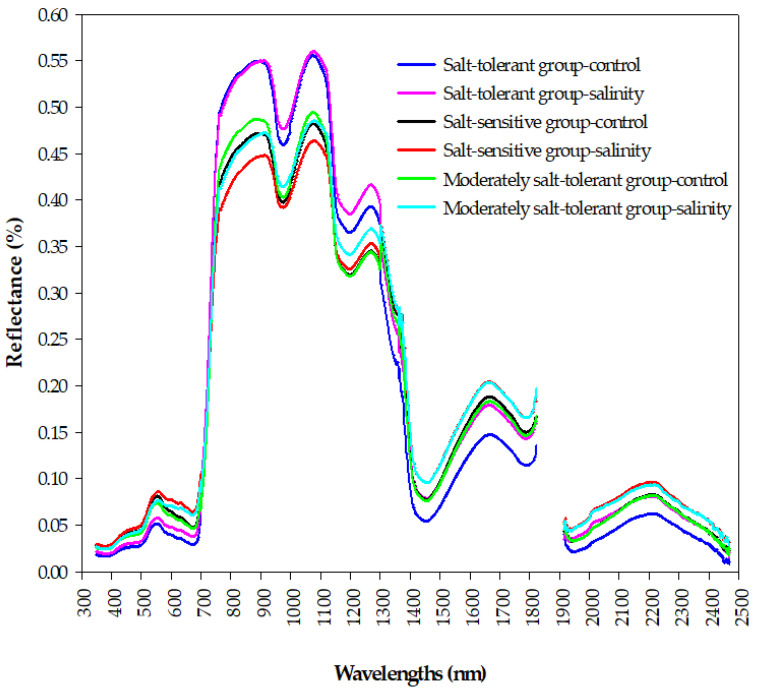
The changes in the shapes of the canopy spectral signatures of the three salinity tolerance groups under the control and salinity conditions in the full spectrum range (350–2500 nm).

Figure 5 shows how the spectral signatures of a canopy depend on the salt tolerance level of wheat genotypes under control and salinity conditions. Generally, the reflectance curves of the three salinity tolerance groups are well-separated from each other at the three main spectrum regions. Within the visible region (VIS, 400–700 nm), the spectral reflectance of the salt-tolerant group under the control and salinity conditions was lower than that of the salt-sensitive and moderately salt-tolerant groups, with an obvious green peak and red valley for the three groups under both conditions (Figure 5). Within the near-infrared region (NIR, 700–1300 nm), the salt-tolerant group showed the highest reflectance values under both conditions, whereas a lower canopy reflectance in this region was found for the salt-sensitive group under salinity conditions. In addition, there was obvious valleys around 960 and 1170 nm for the three groups under both conditions (Figure 5). Within the shortwave-infrared region (SWIR, 1300–2500 nm), the salt-tolerant group under the control condition had a lower canopy reflectance; the opposite held true for salt-sensitive and moderately salt-tolerant groups under the salinity conditions. In addition, the three groups had obvious peaks around 1640 and 2200 nm under the control and salinity conditions (Figure 5). These obvious differences in the spectral signatures between the three groups of salinity tolerance at the three parts of the spectrum provided an optical basis for analyzing and constructing the relationship between the salinity tolerance level of the genotypes and spectral reflectance indices in this study.

### 2.6. Prediction of Grain Yield of the Three Salinity Tolerance Groups under Control and Salinity Conditions by Spectral Reflectance Indices

The relationships between the grain yield and different vegetation and water SRIs were first analyzed by the stepwise multiple linear regression (SMLR) method in order to determine the most effective SRIs that contributed the most variations in the GY under the control and salinity conditions. In general, the vegetation SRIs or water SRIs measured under the control or salinity conditions fitted better with the GYc (R^2^ = 0.64 − 0.79) than GYs (R^2^ = 0.38 − 0.47) (Table 4). The GNDVI, RNDVI, and Chlgreen from the vegetation SRIs and WI, NMDI, and SWSI-1 from the water SRIs were detected by SMLR as the most important indices and explained most of the variations in the GYc and GYs. The Chlgreen and GNDVI measured under control conditions explained 79.0% and 38.0% of the variations in the GYc and GYs, respectively, while, when measured under salinity conditions, they explained 69.0% and 47.0% of the variations in the GYc and GYs, respectively (Table 4). The SWSI-1 measured under salinity conditions explained 64.0% and 42.0% of the variations in the GYc and GYs, respectively, while, when combined with the WI and NDMI and measured under control conditions, it explained 77.0% of the variations in the GYc.

**Figure 6:**
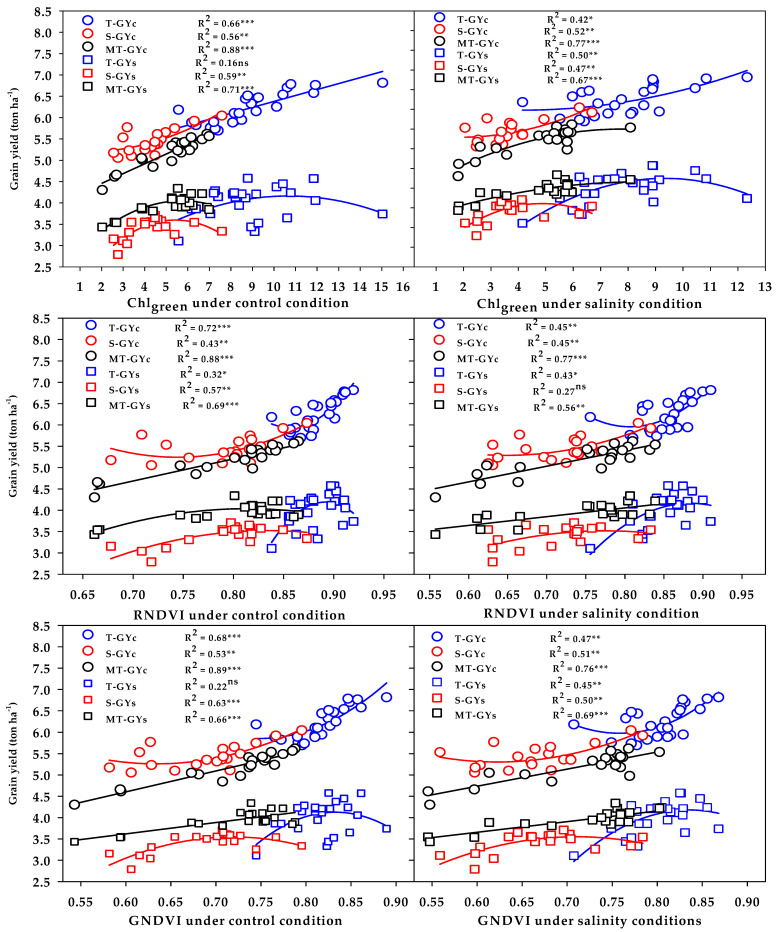
Functional relationship between the selected vegetation SRIs measured under the control and salinity conditions and grain yield under the control (GYc) and salinity (GYc) conditions for the salt-tolerant (T), salt-sensitive (S), and moderately salt-tolerant (MT) genotypes groups. *, **, and *** indicate significance at the 0.05, 0.01, and 0.001 probability levels, respectively, and ns: not significant. The full names of the SRIs are mentioned in Table 6.

**Figure 7:**
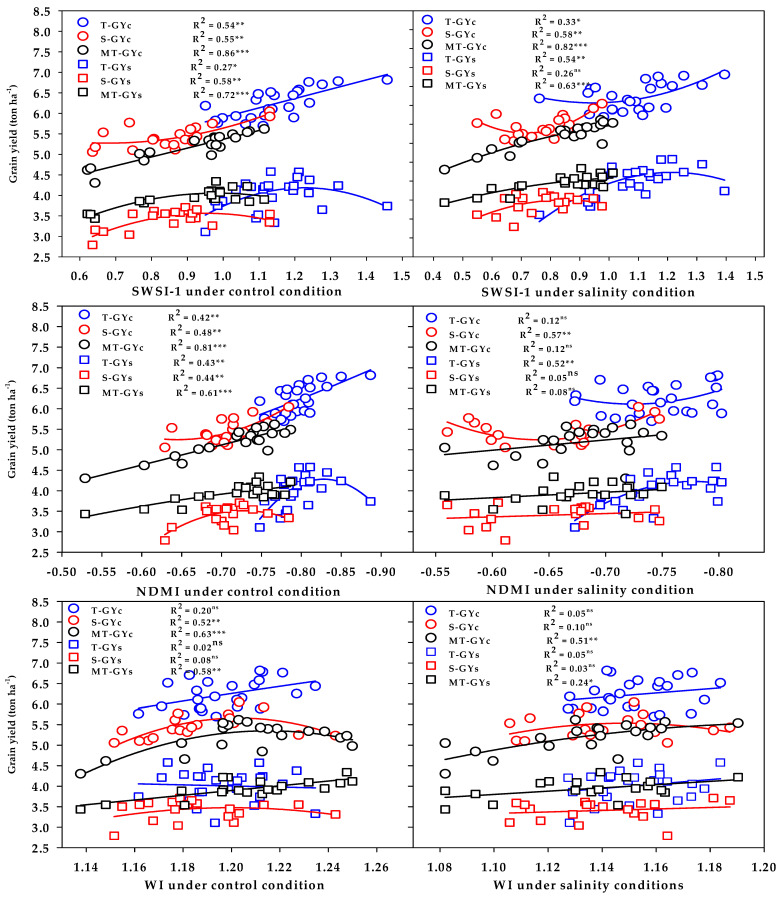
Functional relationship between the selected water SRIs measured under the control and salinity conditions and grain yield under the control (GYc) and salinity (GYc) conditions for the salt-tolerant (T), salt-sensitive (S), and moderately salt-tolerant (MT) genotypes groups. *, **, and *** indicate significance at the 0.05, 0.01, and 0.001 probability levels, respectively, and ns: not significant. The full names of the SRIs are mentioned in Table 6.

The three vegetation SRIs (GNDVI, RNDVI, and Chlgreen) from either the control or salinity treatment showed a strong relationship with GYc (R^2^ = 0.76 − 0.89) and moderate-to-strong relationship with the GYs (R^2^ = 0.56 − 0.71) for the moderately salt-tolerant genotypes group (Figure 6). The three vegetation SRIs from the control treatment showed a strong relationship with the GYc for the salt-tolerant genotypes group (R^2^ = 0.66 − 0.72) and moderate relationship with the GYs for salt-sensitive genotypes group (R^2^ = 0.57 − 0.63); however, when calculated from the salinity treatment, they exhibited moderate relationships with the GYc for the salt-sensitive genotypes group (R^2^ = 0.45 − 0.52), as well as moderate relationships with the GYs for the salt-tolerant genotypes group (R^2^ = 0.43 − 0.50) (Figure 6). The three water SRIs (WI, NDMI, and SWSI-1) from the control treatment showed strong relationships with the GYc (R^2^ = 0.63 − 0.86) and a moderate-to-strong relationship with the GYs (R^2^ = 0.58 − 0.72) for the moderately salt-tolerant genotypes group, whereas they exhibited moderate relationships with the GYc and GYs for the salt-sensitive genotypes group (R^2^ = 0.44 − 0.58) (Figure 7). The SWSI-1 calculated from the salinity treatment was the only index that exhibited a strong relationship with the GYc (R^2^ = 0.82) and GYs (R^2^ = 0.63) for the moderately salt-tolerant genotypes group. The NDMI and SWSI-1 calculated from the salinity treatment showed a moderate relationship with the GYc for the salt-sensitive genotypes group (R^2^ = 0.57 and 0.58) and with the GYs for the salt-tolerant genotypes group (R^2^ = 0.52 and 0.54), respectively (Figure 7).

### 2.7. Prediction of Stress Tolerance Indices of the Three Salinity Tolerance Groups under Control and Salinity Conditions by Spectral Reflectance Indices

Across two salinity treatments and two years, the three vegetation SRIs exhibited a strong relationship with the STI and GMP (R^2^ = 0.61 − 0.89) and a weak-to-moderate relationship with the YSI, SSI, and TOL (R^2^ = 0.13 − 0.51) for the three salinity tolerance groups (Table 5). SWSI-1 was the only index from water SRIs that exhibited a strong relationship with the STI and GMP (R^2^ = 0.63 − 0.91) for the three salt tolerance groups. The NDMI showed the strongest relationships with the STI and GMP (R^2^ = 0.65 − 0.69) for the salt-tolerant and moderately salt-tolerant genotypes groups, whereas the WI showed the strongest relationships with both indices (R^2^ = 0.65 to 0.66) for only the moderately salt-tolerant genotypes group. Three vegetation SRIs and three water SRIs exhibited a weak and nonsignificant relationship with the YSI and SSI for only the moderately salt-tolerant genotypes group, whereas they exhibited weak-to-moderate relationships (R^2^ = 0.25 − 0.51) with both indices for the salt-tolerant and salt-sensitive genotypes groups (Table 5).

## 3. Discussion

In general, the final GY is closely correlated to multiple plant parameters, particularly those related to biomass allocation, as well as the interception and conversion of sunlight. Additionally, almost all of these parameters are formed at key growth stages along the crop growth cycle, so GY represents the entire life of the plants and reflects the extent and magnitude of the negative impacts of environmental stresses to which the plants have been exposed [50,51,52]. Therefore, the GY is considered one of the most targeted traits for evaluating and improving genotypes under both normal and stress conditions in breeding programs. In this study, the mean squares from the ANOVA analysis revealed that there are highly significant differences between the ST and G for the GY in the two years (Table 1), with a wide range between the minimum and maximum values for this trait under the control and salinity conditions (Table 2), which confirms the importance of the GY as an effective screening criterion for evaluating genotypes under both normal and stress conditions. However, because the performances of the genotypes for the GY are not consistent across the normal and stress conditions, several STIs have been proposed as valuable tools for helping breeders to select appropriate genotypes for different environments based on the GY. For instance, high values of the STI indicate a high tolerance to stress with a high yield potential for a given genotype [47]. The YSI was able to identify genotypes that have better GYs under both stress and normal conditions, while the YI was able to select genotypes with a high GY under only stress conditions [44]. The SSI may be useful for isolating susceptible genotypes where the genotypes with high values for this index are more sensitive to stress and vice versa [49]. To identify the genotypes that have the least reduction in GY under stress conditions compared to normal conditions, the TOL may be appropriate in achieving this goal [53]. The GMP is another index with the potential for selecting genotypes that yield well under normal conditions and yield reasonably well under stress conditions [47]. Therefore, these indices provide better opportunities for breeders to select genotypes that do well under a wide range of environmental conditions. In this study, the results of the ANOVA showed highly significant differences between the G for all STIs in the tested years (Table 1), with a wide range between the minimum and maximum values for these indices (Table 2). Importantly, these STIs, along with the GY under both conditions across two years, succeeded in classifying the tested genotypes into three distinct groups according to their level of salt tolerance (Figure 3 and Table 3). These results indicated that the different STIs based on GY can be considered as effective screening criteria when evaluating the salt tolerance of wheat genotypes and could help breeders in identifying genotypes with superior performances under either normal or salinity conditions, as well under both conditions. Similar findings were reported by several studies for several crops under different stressful environments [54,55,56,57,58,59].

However, GY as a screening criterion has a high genotype-by-environment interaction. The yield potential of wheat is very sensitive to various meteorological variables that occur from anthesis to grain filling, which differ from year to year and one environment to another [60,61]. Therefore, repetitive evaluations of genotypes based on GY in different locations for several years are necessary for selecting genotypes that do well under all conditions. Additionally, calculating the different STIs based on the GY requires waiting until the plants reach their maturity stage, as well as evaluating the yield performances of genotypes under both normal and stress conditions. This routine work is time-consuming, cost-inefficient, and labor-intensive, so it makes the evaluation of a large number of genotypes based on the GY and different STIs impractical. Therefore, rapid and cost- and time-efficient tools with early estimations of the GY and STIs are urgently needed, which is of significant importance not only for plant breeders when they evaluate a large number of genotypes under a wide range of environmental conditions but, also, for farmers to manage wheat production under salinity conditions.

### 3.1. Interpreting Canopy Hyperspectral Behavior of Salinity Tolerance Groups under Control and Salinity Conditions

The results of Figure 5 demonstrated that there are clear differences in the shape of canopy spectral reflectance between contrasting salinity tolerance groups under control and salinity conditions in the three main parts of the spectrum (VIS, NIR, and SWIR), which indicate that it is possible to estimate the GY and STIs in terms of their spectral behavior early in a rapid and cost-efficient manner. The salt-tolerant genotypes group, which attained higher values for the GYc, GYs, STI, TOL, and GMP (Table 3), showed lower canopy reflectance in the VIS spectrum and higher canopy reflectance in the NIR spectrum under both conditions, whereas the opposite trend was observed for the salt-sensitive genotypes group, which attained a lower value for the GYs, YSI, STI, and GMP (Table 3), followed by the moderately salt-tolerant genotypes group under salinity conditions. The latter two groups under salinity conditions reflected a higher amount of radiation in the SWIR spectrum, while the opposite trend was observed for the former group under the control conditions (Figure 5). Indeed, the canopy reflectance in the three portions of the VIS spectrum (blue, green, and red) depends mainly on the photosynthetic capacity and content of the different leaf pigments, mainly chlorophyll, carotenoids, flavonoids, and anthocyanin, while canopy reflectance in the NIR region is influenced mainly by the characteristics of the leaf structure, biomass accumulation, and leaf area index, which induce a direct effect on the scattering of light at this region [35,62,63,64]. The canopy reflectance at weak and strong water absorption bands located in NIR and SWIR regions, respectively, are strongly related to the canopy’s water content [28,30,65]. Higher reflectance in the VIS and SWIR regions and lower reflectance in the NIR region for the salt-sensitive genotypes group under salinity condition, suggesting a low amount of leaf pigment contents and photosynthetic capacity, a decrease in plant water content, and significant changes in leaf mesophyll structure and biomass accumulation for genotypes of this group, respectively, and vice-versa for genotypes of salt-tolerant group. These substantial variations in the shape of canopy spectral reflectance in the full-range of the spectrum (VIS, NIR, and SWIR) between contrasting salinity tolerance groups may be attributable to the various effects of different components of salinity stress (osmotic stress, ion toxicities, and ion imbalance) on the different characteristics of canopy such as leaf pigments, leaf structure, dry matter, and plant water status. For example, the buildup of toxic ions (Na^+^ and Cl^−^) in the leaf blade under salinity conditions result in a decrease in contents of chlorophyll and carotenoid and thus more leaf senescence and necrosis which eventually lead to an increase in reflectance in the VIS region, mainly in blue and red regions [62,64,66]. Because K^+^ ion plays a vital role in maintains cell turgor pressure and thereby maintains mesophyll structure and thickness, lower concentrations of K^+^ in the leaf blade under salinity conditions cause changes in leaf mesophyll structure and therefore increase the scattering of light in the NIR region. Additionally, the low water content of the canopy under salinity conditions, which is induced by the osmotic potential of the soil solution due to excess salt concentrations, causes a parallel increase in reflectance at strong water absorption bands located in the SWIR region [30,65]. Because the salt-tolerant genotypes can generate distinct salt tolerance mechanisms to overcome the negative impacts of salinity stress on their different characteristics of canopy compared to salt-sensitive genotypes, this may explain why there are clear differences in the shape of canopy spectral reflectance between the salt-tolerant and salt-sensitive groups in the three parts of the spectrum. Overall, these findings highlight the importance of analyzing the canopy hyperspectral signatures as a rough screening tool for evaluating salinity tolerance of a large number of wheat genotypes in breeding programs. Therefore, in this study, we continued to study the potential of using different SRIs, which incorporate specific wavelengths selected from the three parts of the spectrum, as a proxy tool for the early assessment of GY and STIs.

### 3.2. The Ability of SRIs for Assessment of GY and STIs

Early and accurate estimation of the GY would be useful for plant breeders to reduce the number of crosses in breeding programs as well as for farmers to provide them with a more integrative tool to manage wheat production under a wide range of environmental conditions. The SRIs, which, based on few and specific wavelengths selected from the three parts of the spectrum, offer a simple approach for early estimating the GY, and thus could be used as a fast and cheap screening tool for evaluating genotypes under normal and stress conditions. In this study, we observed significant differences between ST, G, and their interaction for most vegetation and water SRIs (Table 1), with a wide range between the minimum and maximum values for these indices under control and salinity conditions (Table 2). These results confirm that it is possible to estimate the genotypic differences in GY and STIs under control and salinity conditions by the SRIs that combine wavelengths sufficiently sensitive to detect changes in the growth health of plants (i.e., chlorophyll and other pigments content, photosynthetic capacity, leaf structure, leaf area index, and biomass) and/or plant water status (i.e., relative water content, equivalent water thickness, fuel moisture content, and gravimetric water content). This could be due to the fact that, when a large number of genotypes are being evaluated, the wide range of genotypic variability in growth health and/or water status is a logical consideration under both the control and salinity conditions, and thus sufficient genotypic variation in spectral properties at specific wavelengths from VIS, red-edge, NIR, and SWIR regions may exist too under both conditions as confirmed by Figure 5. Previous studies also reported that a large part of the variation in GY and many plant traits related to the growth of plants in different crops under diverse environmental conditions could be explained by several SRIs, which either informing on the growth and/or water status of the plants [15,26,40,41,42,63,67,68,69,70]. However, the potential validity of SRIs in estimating genotypic differences in GY and other plant traits depended on the types of these SRIs (vegetation and water SRIs), phenological growth stages, crop types, and levels of environmental stress. For instance, the SRIs measure the water status of plant and based on wavelengths from red-edge and NIR regions explained a higher proportion of the variability for GY in winter wheat at any individual phenological growth stage compared with the SRIs measure the growth health and based on wavelengths from VIS region [39]. Gutierrez et al. [30] found that the water SRIs performed better than vegetation SRIs in selecting the top-yielding genotypes for GY of wheat under diverse environmental conditions. However, El-Hendawy et al. [42] reported that the vegetation SRIs exhibited strong phenotypic correlation, while the water SRIs exhibited moderate phenotypic correlations with GY of spring wheat under water deficit stress conditions. Most importantly, the ability of SRIs for estimating genotypic differences in GY depended also on the conditions of spectral measurements. For instance, El-Hendawy et al. [68] found that the vegetation SRIs and water SRIs that were calculated from the spectral measurements taken under control conditions exhibited a week relationship with the GY of wheat genotypes for both the control and drought conditions, while they exhibited a strong relationship with the GY of both conditions when they were calculated from the spectral measurements taken under drought condition. The results of this study showed that, in general, seven out of the 10 vegetation SRIs and five out of the 10 water SRIs that were calculated from the spectral measurements taken under control and salinity conditions exhibited strong correlation with GYc, STI, and GMP, moderate correlation with GYs, and weak correlation with TOL, while they failed to correlate with YSI and SSI (Figure 2). These results indicate that it is indeed possible by SRIs to early selecting the genotypes that yielded well under both conditions as well as the genotypes that have yield reasonably well under salinity condition. Additionally, the both vegetation and water SRIs that were calculated from the spectral measurements taken under either control or salinity conditions seem to be effective for estimating genotypic differences in GY of both the control and salinity conditions. Together, these findings indicate that the SRIs are of great practical screening tool for early detecting genotypic differences in GY under both control and salinity conditions. Sufficient differences in morphological characteristics and agronomic traits (i.e., yield components) between genotypes under control conditions, particularly a large number of genotypes were evaluated in this study, might be the primary reasons why the SRIs that were calculated from the spectral measurements taken under control conditions successfully estimated GY. However, the efficiency of SRIs that were calculated from the spectral measurements taken under salinity conditions in estimating GY may be related to genotypic differences in the degree of changes in chlorophyll degradation, anatomical structures of leaf, and water status of leaf induced by different components of salinity stress.

### 3.3. Assessment of GY and STIs for Each Salinity Tolerance Group

The results of SMLR model, which identify the most effective SRIs that explained the most variation in GY across genotypes, reveal that the most efficient SRIs that were selected from either vegetation SRIs or water SRIs and calculated from the spectral measurements taken under control or salinity conditions were much more effective in estimating GY under control conditions (R^2^ = 0.64 − 0.79) than under salinity conditions (R^2^ = 0.38 − 0.47) (Table 4). This finding indicates that the capacity of SRIs to estimate GY were not only highly genotype-dependent but also highly environmental conditions-dependent. In this study, the ability of vegetation and water SRIs that were calculated from the spectral measurements taken under control or salinity conditions to estimate GY was higher in control conditions where genotypes expressed their yield potentiality. This result could be attributed to the wide variation in GY among genotypes under control conditions (GYc ranging from 3.92 to 7.24 in the first year and from 4.24 to 7.81 in the second year; Table 2) compared with those under salinity conditions (GYs ranging from 2.51 to 4.81 in the first year and from 2.27 to 5.21 in the second year; Table 2), which could reflect also a wide variation in morphological diversity among genotypes under control conditions to which the canopy spectral reflectance was captured. Similarly, Royo et al. [71] reported that the capability of some SRIs to estimate the GY of durum wheat increased in the environments that allow genotypes to express their yield potentiality. This is because the wide genotypic differences in GY and other traits related to the growth of plants are usually occurred under favorable conditions and are very limited under severe environmental stress. Ferrio et al. [72] also reported that the ability of SRIs that measured at milk-grain stage to estimate GY of durum wheat was higher in high and medium-yield environmental conditions, but not in low productivity environments.

The results in Table 4 also revealed that the SMLR model identified GNDVI_(940 and 550)_, RNDVI_(990 and 680)_, and Chl_green(760 and 550)_ from vegetation SRIs and WI_(900 and 970)_; NMDI_(860, 1640, and 2130)_; NDMI_(2200 and 1100)_; and SWSI-1_(803, 681, 1326, and 1507)_ from water SRIs as the most efficient SRIs that explained most of the variation in GY among genotypes for both the control and salinity conditions. These results indicate that the SRIs that the wavelengths incorporated within them are effective to detect the alterations that take place in the leaf chlorophyll and other photosynthetic pigment contents, internal leaf structure, biomass accumulation, leaf area index, green area index, and leaf water content can be used as a rapid and non-destructive way for early estimating genotypic differences in GY under control and salinity conditions. Christenson et al. [73] reported that there are some effective wavelengths within the VIS, red-edge, and NIR regions explained much of the variation in GY among soybean genotypes under well-watered and water-stressed conditions, and the green region around 550 nm, the red region around 675 to 695 nm, and the red-edge region from 705 to 745 nm were the best of these wavelengths to estimate GY. Royo et al. [71] also reported that 92% of the variability in the GY among durum wheat genotypes under contrasting Mediterranean conditions being explained by 550 nm. Kawamura et al. [70] found highly significant correlation between GY of six rice cultivars and the reflectance values at 550 nm and 709–711 nm under different transplanting dates. Weber et al. [40] also found that the SRIs that incorporated wavelengths from blue to red regions (495 to 680 nm), from red to red-edge regions (680 to 780 nm), and NIR region (particularly at 900, 970, and 1450 nm) explained most of the variation in GY among maize genotypes under different irrigation water regimes. El-Hendawy et al. [64], reported that about 62%, 74%, 44%, 50%, and 51% of the variability in shoot dry weight of two bread wheat genotypes evaluated under salinity conditions explained by 488 nm, 716 nm, combined 1136 and 1142 nm, 1883 nm, and 2024 nm, respectively. El-Hendawy et al. [22] also reported that the SRIs that incorporated a combination of wavelengths within the green (550 nm), red (650, 670, and 675 nm), red-edge (700, 710, 715, 720, 740, 750, and 780 nm), and NIR (800 and 1100 nm) regions explained 60–81% of the total variability in the content of chlorophyll a, b, and total chlorophyll as well as shoot dry weight of two bread wheat genotypes evaluated under salinity conditions. These findings and our results are an indication of the efficiency of the SRIs that incorporated a combination of wavelengths within the green, red, red-edge, NIR, and SWIR regions for estimating genotypic differences in GY of several field crops under a wide range of environments. Because the degradation of chlorophyll, reduction in biomass, and the alterations in internal leaf structure and leaf water status are real phenomena of osmotic and ion toxicity stresses of salinity, this may be a logical reason explaining why the SRIs that were calculated from the spectral measurements taken under salinity conditions and incorporated a combination of wavelengths within the green, red, red-edge, NIR, and SWIR regions explained most of the variation in GY among genotypes for both the control and salinity conditions. However, the efficiency of SRIs that were calculated from the spectral measurements taken under control conditions could be attributed to the large variability in the photosynthetic and transpiration area of the plant, rate and duration of biomass production, green leaf area duration, and canopy structure and architecture between genotypes that may be sufficient enough to exhibited strong relationships between GY and SRIs that incorporated a combination of wavelengths within the green, red, red-edge, NIR, and SWIR.

The SRIs selected by the SMLR model were used to show the ability of these indices to properly discriminate the three salinity tolerance groups for GY. The results in Figure 6 and Figure 7 reveal that, (1) the vegetation SRIs that were calculated from the spectral measurements taken either under control or salinity conditions performed better than the water SRIs for estimating the GY of both conditions; (2) the relationship between SRIs (either vegetation or water SRIs and either calculated from the spectral measurements taken under control or salinity conditions) and GY of both conditions was higher for the moderately salt-tolerant genotypes group than it was for the salt-tolerant genotypes group and salt-sensitive genotypes group; (3) the SRIs that were calculated from the spectral measurements taken under control conditions enabled an accurate estimation of the GYc of the salt-tolerant genotypes group compared with those calculated from the spectral measurements taken under salinity conditions, while the opposite trend was found for the GYs of the same group; and (4) the SRIs that were calculated from the spectral measurements taken under both control and salinity conditions were comparable in estimating the GYc of the salt-sensitive genotypes group, while the SRIs that were calculated from the spectral measurements taken under salinity conditions enabled an accurate estimation of the GYs of the same group compared with those calculated from the spectral measurements taken under control conditions. All these findings indicate that the efficiency of SRIs for early estimating the GY depend on the type of SRIs, the conditions of spectral measurements, the degree of salt tolerance of genotypes, and the degree of genetic variability in plant characteristics within the genotypes under both control and stress conditions.

Regarding the relationships between SRIs and STIs, the results in Table 5 reveal that, (1) the three vegetation SRIs and SWSI-1 from water SRIs exhibited a strong relationship with STI and GMP for the three salt tolerance groups, and this was evident for the moderately salt-tolerant genotypes group followed by salt-sensitive genotypes group; and (2) the relationship between the three vegetation and water SRIs and the YSI, SSI, and TOL was higher for the salt-sensitive genotypes group than it was for the salt-tolerant genotypes group and the moderately salt-tolerant genotypes group. These findings reveal that it is indeed possible by SRIs to early and properly discriminate the genotypes that have high yield potential accompanied by high tolerance to salt stress and the genotypes that yield well under normal condition and yield reasonably well under salt stress condition, as well as the genotypes that have low yield potential accompanied by a high sensitivity to salt stress. These findings also confirm that the significant variability in plant variables, especially the variables that are sensitive to environmental stress as well as those that show a wide genotypic variability among genotypes under both control and salinity conditions, play a vital role in the ability of SRIs to discriminate the genotypes based on their GY under a wide range of environmental conditions.

## 4. Materials and Methods

### 4.1. Plant Materials and Experimental Setup

A collection of 64 recombinant inbred lines (RILs) and cultivars of spring wheat were used as plant materials in this study. This collection comprised 56 F_8_-RILs and their three parents and five commercial cultivars (Kawz, Gemiza-9, Misr-1, Shandawel-1, and Kharchia 65). The three parents (Sakha 93, Sakha 61, and Sids 1) were previously evaluated and have been identified as salt-tolerant, salt-sensitive, and moderately salt-tolerant cultivars, respectively [43]. The first group of RILs (28 RILs) developed from a cross between Sakha 93 and Sakha 61, while the second one (28 RILs) developed from a cross between Sakha 93 and Sids 1. Gemiza-9, Misr-1, and Shandawel-1 were also previously evaluated under actual saline field conditions and have been ranked as moderately salt-tolerant, moderately salt-sensitive, and salt-sensitive cultivars, respectively [2]. Kharchia 65, which has been used as a standard for the salt tolerance test in several salinity experiments [43,74], was also used as a check cultivar in this study. Therefore, the plant materials used in this study reflected a wide range of genetic variability.

All plant materials were evaluated under control (≈0.35 dS m^−1^) and high salinity levels (15.0 dS m^−1^) during the 2019/2020 and 2020/2021 winter growing seasons at the Experimental Research Station belonging to the College of Food and Agriculture Sciences of the King Saud University, Riyadh, Saudi Arabia (24°25′ N, 46°34′ E; elevation 400 m). The weather at the experimental research station is mostly sunny during the winter growing cycles of wheat (middle of November to the end of April), with the mean precipitation and temperature varying from 4.0 to 20.0 mm and 12.9 to 32.2 °C, respectively. The soil texture is a sandy loam with organic matter; pH; calcium carbonate; and available N, P_2_O_5_, and K_2_O of 0.46%, 7.85, 29.42%, 35.4 ppm, 14.8 ppm, and 243.5 ppm, respectively. Additionally, the soil had a bulk density of 1.48 g cm^−3^, a field capacity of 0.101 m^3^ m^−3^, and water-holding capacity of 0.066 m^3^ m^−3^ [75].

In both years, the experiments were laid out in a randomized complete block design with a split-plot arrangement and three replications. The salinity levels were arranged in the main plots, while the wheat genotypes were randomly arranged in the subplots. Each subplot consisted of five rows 1.5 m long, with a separation of 0.2 m between rows (1.5 m^2^ in total area). Seeds of genotypes were sown on 25 November 2019 and 17 November 2020 at a seeding rate of 15 g m^−2^. Before sowing, the soil was fertilized with 50-kg N ha^−1^, 120-kg P ha^−1^, and 100-kg K ha^−1^. The plants were fertilized again at late tillering, Zadoks stage 28 with 50-kg N ha^−1^ and at late booting, Zadoks stage 47 [76] with 50-kg N ha^−1^ and 50-kg K ha^−1^. The N, P, and K were applied as ammonium nitrate (33.5% N), monocalcium phosphate (15.5% P_2_O_5_), and potassium chloride (60% K_2_O), respectively. Protecting plants from diseases and weeds was done in a timely manner. The genotypes were harvested during the third week of April in both years.

To avoid adverse salinity impacts on the germination and seedling establishment, the salinity treatment was started three weeks after sowing. The genotypes in the salinity treatment were irrigated with artificial saline water containing 8.8-g NaCl L^−1^. In the salinity treatment, the build-up of salt in the root zone was monitored during the growing season through collected soil samples at a depth of 0–60 cm from different places of the main plot. Soil samples were collected four times during the growing cycles of wheat. Based on the average value of the electrical conductivity (EC) analysis for these samples, the EC for the salinity treatment did not exceed 16.3 dS m^−1^ in both years. In the second year, all plots of salinity treatments were irrigated before sowing by freshwater several times to flush the salt from the root zone that accumulated during the first year.

**Figure 8:**
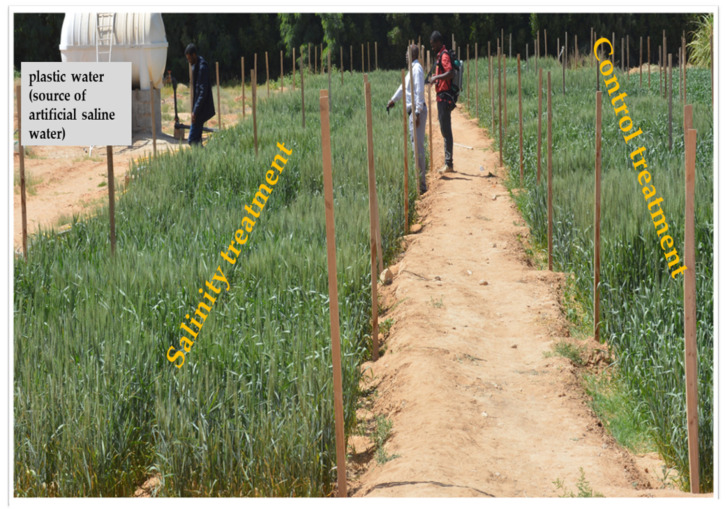
Shown is the overview of the field experiment for the control and salinity treatments.

A low-pressure surface irrigation system was used to apply irrigation water for both treatments. This system consists of a main irrigation line (76 mm in diameter). This line, which delivered saline irrigation water from plastic water tanks (5.0 m^3^) in the salinity treatment (Figure 8) or fresh water from the source of normal water in the control treatment to the subplots, was branched off to the submain hoses at each subplot and equipped with manual control valves to enable controlling the amount of irrigation water delivered to each subplot. In each year, the surface irrigation was applied 10 times for each treatment, with the amount of water totaling 4800 m^3^ ha^−1^.

### 4.2. Grain Yield Measurement and Calculation of Salt Tolerance Indices

Upon reaching maturity, the center two rows of each subplot harvested by hand, threshed, and GY were recorded and expressed as ton per hectare after being adjusted to a 14% moisture content. Based on the GY of the control (GYc) and salinity (GYs) treatments for each genotype, different STIs were calculated. The equations of these indices are listed with their references in Table 6.

### 4.3. Spectroradiometric Data and Processing

The data of the field spectrum reflected from the canopy of each genotype under both the control and salinity treatments were collected at the mid-anthesis growth stage, Zadoks stage 65, using the ASD Field Spec 4 Standard-Res portable spectroradiometer (Analytical Spectral Devices Inc., Boulder, Colorado, CO, USA). This device detects the light scattered by a canopy in the optical range between 350 and 2500 nm, with spectral intervals of 3 nm and 10 nm within spectral regions 350–1000 nm (VIS-NIR region) and 1000–2500 nm (NIR-SWIR region), respectively. However, the spectral intervals in the full spectrum range were finally calculated automatically to achieve 1.0-nm-width continuous bands (2150 continuous bands). The light reflected from the canopy is captured by a fiber optic cable, which was constrained in this study by a 25° field of view.

(FOV) fore-optic. To minimize the impacts of the canopy shadow and sun angle on spectral measurements, the fore-optic was held approximately 1.0 m above the canopy in the nadir direction, and the measurements were made on a sunny day between 11:00 and 15:00 h and calibrated using a Spectralon (Spectralon Labs, ASD) white reference panel (white barium sulfate), which was made immediately before canopy spectral measurements for each subplot. The final reflectance spectrum was calculated as the ratio between the reflected light from the canopy against the total radiance reflectance from the surface of the white reference panel. Two sequential spectral measurements were taken per subplot with a scanning area of approximately 0.20 m^2^ in the center of each measurement. An average of two measurements and 10 scans for each was recorded as the measured spectrum per subplot and used to calculate different SRIs. These SRIs were selected to cover 10 vegetation SRIs, which related directly to the status of the growth vigor and to changes in the photosynthetic efficiency, pigment contents, and aboveground biomass, and 10 water SRIs, which related to changes in the internal leaf structure, leaf biochemical compounds, and canopy water status. All of these plant characteristics are already influenced by salinity stress. The names, abbreviations, and equations of these SRIs are listed with their references in Table 6.

Due to strong absorption/scatter wavebands not related to the canopy reflectance and strongly affected by the atmospheric vapor and carbon dioxide, the wavebands from 1825 to 1915 nm and 2470 to 2500 nm were omitted in this study before calculating the different SRIs.

### 4.4. Data Analysis

The impacts of the salinity treatments (ST), genotypes (G), and their interactions on the GY, STIs, and SRIs were examined in each individual year and combined two years using an appropriate analysis of variance (ANOVA). The data of the GY and SRIs were subjected to ANOVA appropriate for a randomized complete block split-plot design, while the data of calculated STIs for the genotypes were subjected to ANOVA appropriate for a randomized complete block design. The ST, G, and their interactions were considered as fixed effects, whereas years, replications, and their interactions were considered as random effects.

PROC MIXED following the type 1 method was used to obtain mean squares of the combined analysis. Pearson’s correlation coefficients (r) were used to estimate the relationships of the GY and STIs with different SRIs that were calculated from the spectral measurements taken under both the control and salinity treatments for each year and across two years.

The different genotypes were clustered into three salinity tolerance groups (salt-tolerant, salt-sensitive, and moderately tolerant groups) based on the GYc, GYs, and the five STIs across two years using Ward’s minimum variance cluster method.

To identify the most important SRIs accounting the most variability in the GY and STIs, the different vegetation SRIs and water SRIs were further analyzed using the stepwise multiple linear regression (SMLR) method, with the GY and STIs were considered as dependent variables and different SRIs as independent variables. The different models of the best SRIs of each vegetation SRI and water SRI were used to predict the GY and STIs for each salinity tolerance group. The model with the highest values of coefficients of determination (R^2^) was designated the model with the higher prediction accuracy.

## 5. Conclusions

The results of this study found that it is possible to identify genotypes that have a high yield potential accompanied by a high tolerance to salt stress or vice versa, as well as genotypes that produce a desirable yield in both the control and salinity conditions through different STIs. These STIs, along with the GY under the control and salinity conditions, succeeded in classifying the tested genotypes into three distinct groups according to their level of salt tolerance, which indicated the potential use of these indices and GYs as effective screening criteria for discriminating the salt tolerance among wheat genotypes. However, there is a pressing need to early estimates of these screening criteria for a large number of genotypes in a fast and nondestructive manner to accelerate the development of genotypes for salinity stress conditions. The results of this study clearly demonstrated the potential of using SRIs as a fast and cheap screening tool in breeding programs for early estimating of the GYs and STIs. However, the efficiency of these SRIs for simultaneously assessing the production and salinity tolerance of genotypes under a wide range of environmental conditions depends on the type of SRIs, the conditions of the spectral measurements, the degree of salt tolerance of the genotypes, and the degree of genetic variability in the plant characteristics within the genotypes.

## Figures and Tables

**Table 1:** Mean squares for the effects of the salinity treatment (ST), genotypes (G), year (Y), and their possible interactions by ANOVA on the grain yield (GY), different spectral reflectance indices (SRIs), and stress tolerance indices (STIs) for each growing year and combined two years.

	2019–2020			2020–2021			Combined Two Years						
Effect	ST	G	ST × G	ST	G	ST × G	Y	ST	ST × Y	G	G × Y	ST × G	ST × G × Y
DF	1	63	63	1	63	63	1	1	1	63	63	63	63
GY	333.5 ***	1.01 ***	0.605 ***	333.2 ***	1.05 ***	0.526 ***	14.09 ^ns^	666.7 ***	0.002 ^ns^	1.85 ***	0.219 **	1.05 ***	0.082 ^ns^
Vegetation SRIs													
NDVI-1	0.864 ***	0.013 ***	0.012 ***	0.117 *	0.015 ***	0.008 ***	0.693 *	0.808 ***	0.173 ***	0.018 ***	0.010 ***	0.010 ***	0.011 ***
NDVI-2	0.686 ***	0.010 ***	0.010 ***	0.056 ^ns^	0.013 ***	0.008 ***	0.314 *	0.566 ***	0.175 **	0.014 ***	0.009 ***	0.008 ***	0.010 ***
BNDVI	0.004 ^ns^	0.010 ***	0.009 ***	0.021 *	0.012 ***	0.001 ***	0.022 *	0.003 *	0.022 ***	0.021 ***	0.001 ***	0.009 ***	0.001 ***
GNDVI	0.034 *	0.040 ***	0.002 ***	0.009 ^ns^	0.037 ***	0.003 ***	0.007 ^ns^	0.040 **	0.004 ^ns^	0.074 ***	0.003 ***	0.002 ***	0.002 ***
RNDVI	0.384 **	0.045 ***	0.004 ***	0.143 **	0.024 ***	0.004 ***	0.726 ***	0.498 ***	0.029 *	0.064 ***	0.005 ***	0.005 ***	0.003 ***
Chl_green_	60.6 **	33.4 ***	1.25 ***	42.6 *	42.8 ***	4.17 ***	115.5 **	102.4 ***	0.794 ^ns^	73.6 ***	2.56 ***	2.97 ***	2.45 ***
Chl_red-edge_	90.7 ***	1.27 ***	1.26 ***	8.16 ^ns^	2.10 ***	1.12 ***	71.91 *	76.62 ***	22.22 **	1.97 ***	1.39 ***	1.11 ***	1.27 ***
EVI	2.47 **	0.090 ***	0.010 ***	0.052 ^ns^	0.050 ***	0.009 ***	0.572 **	0.905 ***	1.62 ***	0.130 ***	0.010 ***	0.011 ***	0.007 ***
MTVI	2.61 **	0.084 ***	0.010 ***	0.234 *	0.048 ***	0.008 **	0.037 ^ns^	0.640 ***	2.20 ***	0.122 ***	0.009 ***	0.011 ***	0.007 ***
OSAVI	0.714 **	0.051 ***	0.004 ***	0.021 ^ns^	0.027 ***	0.004 ***	0.410 **	0.490 ***	0.245 ***	0.074 ***	0.005 ***	0.005 ***	0.003 ***
Water SRIs													
WI	0.194 ***	0.003 ***	0.003 ***	0.383 **	0.003 ***	0.003 ***	0.518 **	0.562 ***	0.016 *	0.004 ***	0.002 ***	0.003 ***	0.003 ***
NWI-1	0.063 **	0.002 ***	0.002 ***	0.147 **	0.009 ***	0.007 ***	0.397 ***	0.202 ***	0.009 *	0.002 ***	0.001 ***	0.001 ***	0.001 ***
NWI-2	0.037 ***	0.006 ***	0.006 ***	0.066 **	0.005 ***	0.005 ***	0.093 **	0.101 ***	0.002 ^ns^	0.007 ***	0.004 ***	0.006 ***	0.003 ***
WBI	1.32 **	0.031 ***	0.024 ***	0.226 ^ns^	0.022 *	0.017 ^ns^	3.12 **	1.32 ***	0.226 *	0.035 ***	0.018 **	0.021 ***	0.021 ***
NDWI	0.465 **	0.023 ***	0.008 ***	0.080 *	0.019 ***	0.005 ***	0.909 **	0.465 ***	0.080 **	0.038 ***	0.004 ***	0.008 ***	0.005 ***
NDMI	0.470 **	0.022 ***	0.008 ***	0.049 *	0.021 ***	0.006 ***	0.680 **	0.411 ***	0.108 **	0.038 ***	0.004 ***	0.008 ***	0.006 ***
DMCI	0.015 *	0.001 ***	0.002 ***	0.077 ^ns^	0.005 ***	0.005 ***	0.087 *	0.079 *	0.012 ^ns^	0.004 ***	0.003 ***	0.004 ***	0.003 ***
NMDI	0.087 *	0.027 ***	0.005 ***	0.145 **	0.030 ***	0.007 ***	0.213 *	0.229 ***	0.004 ^ns^	0.052 ***	0.005 ***	0.005 ***	0.007 ***
SWSI-1	1.55 *	0.243 ***	0.022 ***	0.048 ^ns^	0.194 ***	0.023 ^ns^	0.796 *	1.07 ***	0.529 **	0.415 ***	0.022 ***	0.024 ***	0.021 ***
SWSI-2	0.036 ^ns^	0.211 ***	0.054 ***	6.04 **	0.105 ***	0.048 ***	2.82 *	2.57 ***	3.51 ***	0.264 ***	0.052 ***	0.051 ***	0.051 ***
Stress tolerance indices (STIs)													
YSI		0.023 ***			0.021 ***		0.017 ^ns^			0.040 ***	0.004 ***		
SSI		0.199 ***			0.206 ***		0.005 *			0.372 ***	0.033 **		
STI		0.040 ***			0.045 ***		0.028 ^ns^			0.075 ***	0.009 ^ns^		
TOL		1.21 ***			1.05 ***		0.005 ^ns^			2.10 ***	0.164 *		
GMP		0.460 ***			0.540 ***		7.28 ^ns^			0.885 ***	0.115 ^ns^		

*, **, and *** indicate significance at *p* ≤ 0.05, 0.01, and 0.001, respectively, and ns indicates not significant. The full names of the different SRIs and STIs are listed in Table 6.

**Table 2:** Statistical parameters (minimum (Min), maximum (Max), and mean values) of all the tested genotypes for the grain yield (GY), different spectral reflectance indices (SRIs), and stress tolerance indices (STIs) under the, control and salinity treatments during two growing years. Data is the average of three replications.

Traits	2019–2020						2020–2021					
	Control			Salinity			Control			Salinity		
	Min	Max	Mean	Min	Max	Mean	Min	Max	Mean	Min	Max	Mean
GY (ton ha^−1^)	3.92	7.24	5.54	2.51	4.81	3.67	4.24	7.81	5.81	2.27	5.21	3.94
Vegetation SRIs												
NDVI-1	0.496	0.769	0.633	0.276	0.727	0.538	0.387	0.776	0.663	0.340	0.767	0.628
NDVI-2	0.429	0.706	0.563	0.271	0.650	0.478	0.320	0.698	0.573	0.305	0.698	0.549
BNDVI	0.720	0.945	0.871	0.711	0.960	0.878	0.704	0.947	0.871	0.658	0.944	0.856
GNDVI	0.485	0.901	0.747	0.412	0.876	0.728	0.532	0.906	0.749	0.467	0.885	0.739
RNDVI	0.487	0.931	0.803	0.391	0.900	0.740	0.666	0.950	0.852	0.542	0.936	0.813
Chl_green_	1.297	16.867	6.023	0.926	13.152	5.228	2.088	17.532	6.734	1.357	14.048	6.068
Chl_red-edge_	1.828	5.874	3.202	0.752	4.650	2.230	1.099	5.860	3.474	0.958	5.866	3.182
EVI	0.321	0.972	0.751	0.187	0.920	0.591	0.507	0.917	0.714	0.385	1.007	0.737
MTVI	0.317	0.962	0.725	0.151	0.898	0.560	0.430	0.861	0.632	0.339	0.983	0.681
OSAVI	0.415	0.887	0.742	0.292	0.849	0.655	0.558	0.873	0.752	0.463	0.879	0.737
Water SRIs												
WI	1.071	1.300	1.167	1.016	1.202	1.122	1.126	1.321	1.228	1.054	1.258	1.164
NWI-1	−0.116	0.043	−0.039	−0.070	0.081	−0.013	−0.132	−0.040	−0.091	−0.094	0.014	−0.052
NWI-2	−0.130	−0.034	−0.077	−0.092	−0.008	−0.057	−0.138	−0.059	−0.102	−0.114	−0.026	−0.076
WBI	−0.095	0.514	0.207	−0.001	0.628	0.324	−0.246	0.374	0.114	−0.189	0.566	0.163
NDWI	0.484	0.887	0.750	0.460	0.837	0.681	0.534	0.936	0.799	0.514	0.910	0.770
NDMI	−0.872	−0.464	−0.726	−0.810	−0.446	−0.656	−0.917	−0.535	−0.762	−0.882	−0.455	−0.739
DMCI	−0.263	−0.088	−0.187	−0.326	−0.112	−0.200	−0.388	−0.035	−0.201	−0.484	−0.109	−0.229
NMDI	0.314	0.816	0.645	0.443	0.795	0.615	0.442	0.835	0.683	0.091	0.828	0.644
SWSI-1	0.363	1.474	0.980	0.308	1.463	0.853	0.525	1.571	0.992	0.419	1.654	0.970
SWSI-2	0.854	2.242	1.681	0.884	2.280	1.662	1.078	1.760	1.425	1.127	2.439	1.676
Stress tolerance indices (STIs)												
	2019–2020						2020–2021					
YSI	0.446	0.869	0.670				0.429	0.857	0.683			
SSI	0.399	1.621	0.979				0.439	1.790	0.987			
STI	0.395	1.054	0.667				0.319	1.193	0.684			
TOL	0.593	3.437	1.864				0.707	3.728	1.863			
GMP	3.481	5.696	4.497				3.196	6.376	4.772			

The full names of the different SRIs and STIs are listed in Table 6.

**Table 3:** Mean values of the grain yield under the control and salinity conditions, and different stress tolerance indices of the three clusters group. The values averaged over the two years.

Traits	Salt-Tolerant Group	Salt-Sensitive Group	Moderately Salt-Tolerant Group
Number of genotypes in each cluster	25	19	20
Grain yield under control condition (GYc, ton ha^−1^)	6.23	5.45	5.18
Grain yield under salinity condition (GYs, ton ha^−1^)	4.01	3.41	3.93
Yield stability index (YSI)	0.65	0.63	0.76
Stress susceptibility index (SSI)	1.07	1.13	0.73
Stress tolerance index (STI)	0.78	0.58	0.64
Tolerance index (TOL)	2.22	2.04	1.25
Geometric mean productivity (GMP)	4.99	4.30	4.51

**Table 4:** Selection of the most important spectral reflectance indices (SRIs) for assessment of the grain yield under the control (GYc) and salinity (GYs) conditions based on a stepwise multiple linear regression analysis. The estimates were calculated across two years.

Treatments	Equation	R^2^	RMSE
Vegetation SRIs			
Control	GY_C_ = 4.456 + 0.190_(Chlgreen)_	0.79	0.266
	GY_S_ = 1.446 + 3.155_(GNDVI)_	0.38	0.317
Salinity	GY_C_ = 3.167 + 2.372_(RNDVI)_ + 0.117_(Chlgreen)_	0.69	0.321
	GY_S_ = 1.355 + 3.341_(GNDVI)_	0.47	0.292
Water SRIs			
Control	GY_C_ = 6.037 − 4.07_(WI)_ − 3.91_(NDMI)_ + 1.62_(SWSI-1)_	0.77	0.279
	GY_S_ = 1.457 + 3.539_(NMDI)_	0.38	0.316
Salinity	GY_C_ = 3.574 + 2.299_(SWSI-1)_	0.64	0.345
	GY_S_ = 2.616 + 1.307_(SWSI-1)_	0.42	0.305

R^2^ and RMSE indicate coefficients of determination and root mean squared errors, respectively. The full names of the SRIs are mentioned in Table 6.

**Table 5:** The best models of the regression and determination coefficients (R^2^) for the relationships between the selected vegetation SRIs and water SRIs and different stress tolerance indices (STIs) across two salinity treatments and two years for the three salinity tolerance groups. L and Q indicate linear and quadratic fitting models, respectively.

STIs	Salt Tolerance Groups	Vegetation SRIs			Water SRIs		
		GNDVI	RNDVI	Chl_green_	WI	NDMI	SWSI-1
YSI	Salt-tolerant group	0.25 * Q	0.30 * Q	0.17 ^ns^ Q	0.14 ^ns^ Q	0.43 ** Q	0.33 * Q
	Salt-sensitive group	0.49 ** Q	0.51 ** Q	0.41 ** Q	0.29 * Q	0.48 ** Q	0.47 ** Q
	Moderately salt-tolerant group	0.13 ^ns^ Q	0.21 ^ns^ Q	0.13 ^ns^ Q	0.09 ^ns^ Q	0.09 ^ns^ Q	0.16 ^ns^ Q
SSI	Salt-tolerant group	0.25 * Q	0.30 * Q	0.17 ^ns^ Q	0.14 ^ns^ Q	0.43 ** Q	0.33 * Q
	Salt-sensitive group	0.49 ** Q	0.51 ** Q	0.41 ** Q	0.29 * Q	0.48 ** Q	0.47 ** Q
	Moderately salt-tolerant group	0.13 ^ns^ Q	0.21 ^ns^ Q	0.13 ^ns^ Q	0.09 ^ns^ Q	0.09 ^ns^ Q	0.16 ^ns^ Q
STI	Salt-tolerant group	0.61 *** Q	0.65 *** Q	0.65 *** Q	0.33 * Q	0.65 *** Q	0.71 *** Q
	Salt-sensitive group	0.75 *** Q	0.78 *** Q	0.78 *** Q	0.15 ^ns^ L	0.41 ** Q	0.66 *** Q
	Moderately salt-tolerant group	0.88 *** L	0.88 *** Q	0.88 *** Q	0.65 *** L	0.69 *** Q	0.90 *** Q
TOL	Salt-tolerant group	0.27 * Q	0.31 * Q	0.19 ^ns^ Q	0.14 ^ns^ Q	0.34 * Q	0.30 * Q
	Salt-sensitive group	0.46 ** Q	0.46 ** Q	0.41 ** Q	0.32 * Q	0.50 ** Q	0.49 ** Q
	Moderately salt-tolerant group	0.32 * Q	0.39 * Q	0.32 * Q	0.19 ^ns^ Q	0.25 * L	0.36 * Q
GMP	Salt-tolerant group	0.62 *** Q	0.67 *** Q	0.65 *** Q	0.33 * Q	0.67 *** Q	0.72 *** Q
	Salt-sensitive group	0.71 *** Q	0.75 *** Q	0.75 *** Q	0.15 ^ns^ Q	0.41 ** Q	0.63 *** Q
	Moderately salt-tolerant group	0.89 *** L	0.89 *** Q	0.89 *** Q	0.66 *** Q	0.69 *** Q	0.91 *** Q

*, **, and *** indicate significance at the 0.05, 0.01, and 0.001 probability levels, respectively, and ns: not significant. The full names of the SRIs and STIs are mentioned in Table 6.

**Table 6:** The full names, abbreviations, equations, and references of the stress tolerance indices (STIs) and spectral reflectance indices (SRIs) used in this study.

Different Indices	Equation	Ref.
STIs		
Yield stability index (YSI)	YSI = GY_s_/GY_c_	[77]
Stress susceptibility index (SSI)	SSI = (1− GY_s_/GY_c_)/(1 − GÝ_s_/ GÝ_c_)	[49]
Stress tolerance index (STI)	STI = (GY_c_ × GY_s_)/(GYs)	[47]
Tolerance index (TOL)	TOL = GY_c_ − GY_s_	[47]
Geometric mean productivity (GMP)	GMP = (GY_c_ × GY_s_)	[47]
Vegetation SRIs		
Normalized difference vegetation index (NDVI-1)	(R_750_ − R_705_)/(R_750_ + R_705_)	[78]
Normalized difference vegetation index (NDVI-2)	(R_780_ − R_715_)/(R_780_ + R_715_)	[19]
Blue normalized difference vegetation index (BNDVI)	(R_970_ − R_420_)/(R_970_ + R_420_)	[19]
Green normalized difference vegetation index (GNDVI)	(R_940_ − R_550_)/(R_940_ + R_550_)	[73]
Red normalized difference vegetation index (RNDVI)	(R_990_ − R_680_)/(R_990_ + R_680_)	[79]
Green chlorophyll index (Chl_green_)	(R_760_/R_550_) − 1	[79]
Red edge chlorophyll index (Chl_red-edge_)	(R_760_/R_710_) − 1	[19]
Enhanced vegetation index (EVI)	2.5 [(R_782_ − R_675_)/(R_782_ + 6 × R_675_ − 7.5 × R_445_ + 1)]	[80]
Modified Transformed Vegetation Index (MTVI)	1.2 × [(1.2 × (R_800_ − R_550_) − 2.5 × (R_670_ − R_550_)]	[81]
Optimized soil adjusted vegetation index (OSAVI)	1.16 × (R_800_ − R_670_)/(R_800_ + R_670_ + 0.16)	[82]
Water SRIs		
Water index (WI)	(R_900_/R_970_)	[83]
Normalized water index -1 (NWI-1)	(R_970_ − R_880_)/(R_970_ + R_880_)	[37]
Normalized water index -2 (NWI-2)	(R_970_ − R_900_)/(R_970_ + R_900_)	[39]
Water balance index (WBI)	(R_1500_ − R_531_)/(R_1500_ + R_531_)	[32]
Normalized difference water index (NDWI)	(R_860_ − R_2270_)/(R_860_ + R_2270_)	[19]
Normalized difference moisture index (NDMI)	(R_2200_ − R_1100_)/(R_2200_ + R_1100_)	[84]
Dry matter content index (DMCI)	(R_2305_ − R_1495_)/(R_2305_ + R_1495_)	[85]
Normalized multi-band drought index (NMDI)	860 − (R_1640_ − R_2130_)/860 + (R_1640_ − R_2130_)	[86]
Salinity and water stress index-1 (SWSI-1)	(R_803_ − R_681_)/√(R_1326_ − R_1507_)	[87]
Salinity and water stress index-2 (SWSI-2)	(R_803_ − R_681_)/√(R_905_ − R_972_)	[87]

GY_c_ and GY_s_ are the grain yields of wheat genotypes grown under the control and salinity conditions, respectively. GÝ_c_ and GÝ_s_ are the mean grain yields of all wheat genotypes under the control and salinity conditions, respectively.

## Data Availability

All data are presented within the article.
